# Effect of berberine chloride on caspase-3 dependent apoptosis and antioxidant capacity in the hippocampus of the chronic cerebral hypoperfusion rat model

**DOI:** 10.22038/ijbms.2018.31225.7534

**Published:** 2019-02

**Authors:** Zeynab Pirmoradi, Mayam Yadegari, Ali Moradi, Fatemeh Khojasteh, Fatemeh Zare Mehrjerdi

**Affiliations:** 1 Neurobiomedical Research Center, School of Medicine, Shahid Sadoughi University of Medical Sciences, Yazd, Iran; 2 Deptartment of Anatomy and Cell Biology, School of Medicine, Shahid Sadoughi University of Medical Sciences, Yazd, Iran; 3 Department of Biochemistry, School of Medicine, Shahid Sadoughi University of Medical Sciences, Yazd, Iran; 4 Neuroscience Research Center, Iran University of Medical Sciences, Tehran, Iran

**Keywords:** Antioxidant enzymes, Apoptosis, Berberine, Chronic cerebral – hypoperfusion, Common carotid artery MDA, Rat

## Abstract

**Objective(s):**

The main goal of the current research was to examine the effects of Berberine (BBR) on apoptotic signaling and hippocampal oxidative stress induced by common carotid artery occlusion.

**Materials and Methods:**

Chronic cerebral hypoperfusion (CCH) model was created by occluding the two common carotid arteries (two-vessel occlusion [2VO]) permanently. BBR (50 and 100 mg/kg/daily) was intra-gastrically administered to ischemic rats. Neuronal survival was evaluated by Nissl staining. The levels of malondialdehyde (MDA) and antioxidant enzymes, including catalase (CAT) and superoxide dismutase (SOD), along with the activities of caspase 3 were estimated in the hippocampus 2 month after treating the rats with 2VO.

**Results:**

According to findings of the present research, the BBR therapy inhibited the neuro-degeneration of hippocampus. BBR also significantly decreased the amount of MDA and activity of caspase 3 in the hippocampus. Furthermore, the administration of BBR alleviated the lowered activities of SOD and CAT after 2VO surgery.

**Conclusion:**

The antioxidant and antiapoptotic properties of BBR might play important roles in improving functional outcomes and might have significant neuroprotective effects on the CCH damage.

## Introduction

Cerebral ischemia is the most prevalent cause of disability, as well as the most important cause of mortality, especially in the developed countries. There are several types of ischemia such as the acute brain ischemia, which is classified in two types of global and focal ischemia (1). Focal cerebral ischemia happens when the blood flow is reduced in a particular area of brain. Symptoms are different depending on the region of damaged brain (2). Global brain ischemia is a disorder, where the entire blood flow to the brain is stopped or reduced (1). This is commonly triggered by cardiac arrest. Chronic cerebral hypoperfusion (CCH) is a condition in which the flow of blood toward the brain mildly reduces for a long time (3). It is revealed that the CCH is a pathophysiological mechanism that is both conventional and fundamental, and is regarded as a main factor for cognitive deterioration and degenerative procedures that results in dementia (3, 4). CCH is also an etiological determinant of Alzheimer’s disease (AD) (4). The hippocampal region is one of the main cerebral areas, which performs a notable role on learning and memory. This part of the brain, especially the CA1 pyramidal neurons, is very vulnerable to ischemic insults (5). Various types of cerebral ischemia  have almost the same harmful mechanisms. Increased oxidative stress is a vital hypothesis about the origin of neurological impairment by vascular events (6, 7). 

A major evidence has suggested a connection between an increased creation of reactive oxygen species (ROS) and the expansion of neuronal mortality in different neurological problems, such as epilepsy, Alzheimer’s, brain ischemia and trauma (8). In addition, it was indicated that the higher oxidative stress during the brain ischemia might stimulate the beta-amyloid plaques formation. This probably indicated the association between AD and stroke (9). Severe oxidative stress during the cerebral ischemia can lead to the cell mortality by a necrotic or an apoptotic pathway. Consequently, antioxidants are able to decrease the brain impairment followed by ischemia (8, 10). 

Berberine (BBR) is the principal active compound, originally separated from therapeutic plants, including *Phellodendri sp*., *Coptidis sp*., and *Berberis sp*., with an extended past in the traditional Indian and Chinese medicine (11). Numerous researches have demonstrated several pharmacological and biochemical activities of BBR including the anti-inflammatory, anti-cancer, anti-diabetic and anti-diarrhea actions (11). The obtained data from previous experiments also indicated that BBR might be used as a useful antioxidant in a variety of pathological conditions (12). It has also been revealed that the BBR produces neuroprotective effects through its anti-inflammatory, antioxidative and anti-neuronal apoptosis pharmacological properties on the brain ischemia (12, 13). 

As such, the current research was first planned to evaluate the neuroprotective effects of BBR on the CCH that induced histological and biochemical alternation in hippocampus of rats.

## Materials and Methods

***Animals***

Forty adult male Wistar rats weighting 200-250 g were used in this experiment (10 rats in each group). All rats were caged in a room with a monitored humidity of 55 ± 5% and temperature of 24 ± 1°C with a typical 12-hr light/dark cycle and were allowed to consume liberal amounts of food and water. All phases of the experiment were approved by the Ethics Committee of Yazd University of Medical Sciences and in line with the US National Institutes of Health Publication guidance for the maintenance and application of laboratory animals. All attempts were made to minimize the animals suffering. The rats were adjusted to the housing circumstances for seven days before experiment. The rats were randomly divided into four groups as follow: The CCH group, which were exposed to stable common carotid arteries occlusion, the sham group underwent the same process with no arterial ligation, the BBR treatment groups were treated with 50 and 100 mg/kg BBR (14) by intra-gastric administration once a day for 2 month starting from surgery (Berberine hydrochloride, (Sigma-Aldrich Co), which was dissolved in normal saline (14).

**Figure 1:**
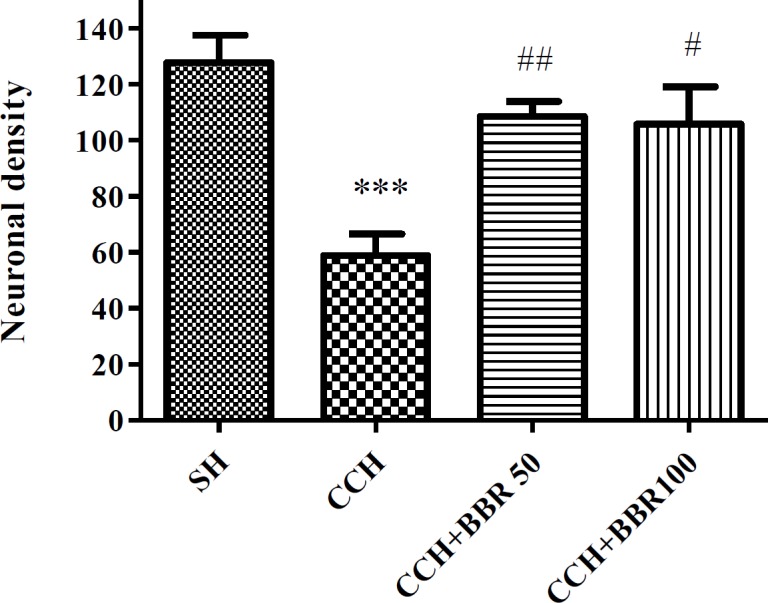
The effects of berberine (BBR) on neuronal density in the hippocampus of the studied groups. Values represent means±SEM. ***P<0.001 compared to sham (SH) group. ##*P*<0.01, #*P*<0.05 compared to chronic cerebral hypoperfusion (CCH) group

**Figure 2:**
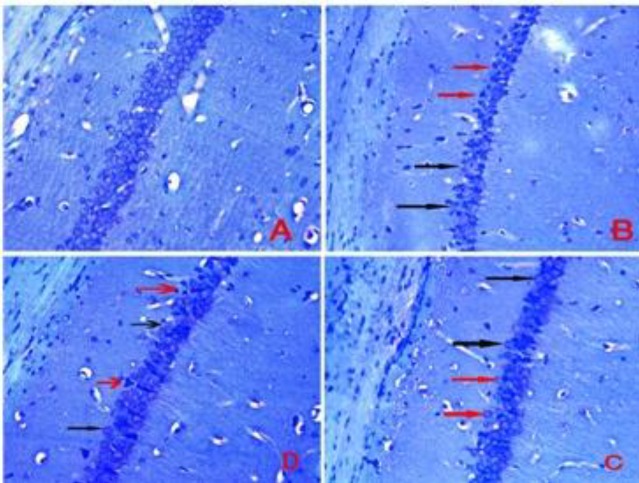
Nissl staining of hippocampal CA1 region. A: sham (SH) group, B: chronic cerebral hypoperfusion (CCH) group, C: CCH +berberine (BBR) 50 group, D: CCH+BBR 100 group. The black arrows indicate intact cells and red arrows indicate necrotic cells (magnification ×400)

**Figure 3:**
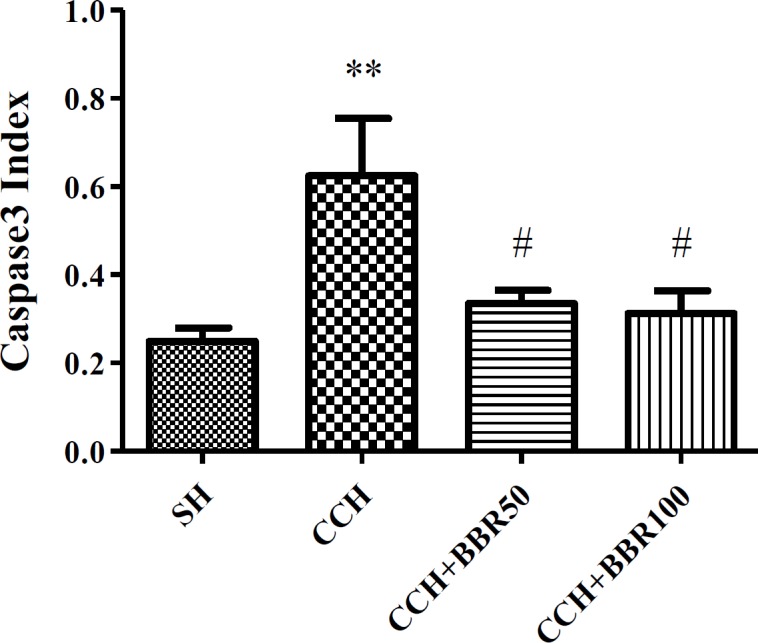
The effects of berberine (BBR) on activity of caspase 3 in the hippocampus of the studied groups. Values represent means±SEM. **P<0.01 compared to sham group. #*P*<0.05 compared to chronic cerebral hypoperfusion (CCH) group

**Figure 4:**
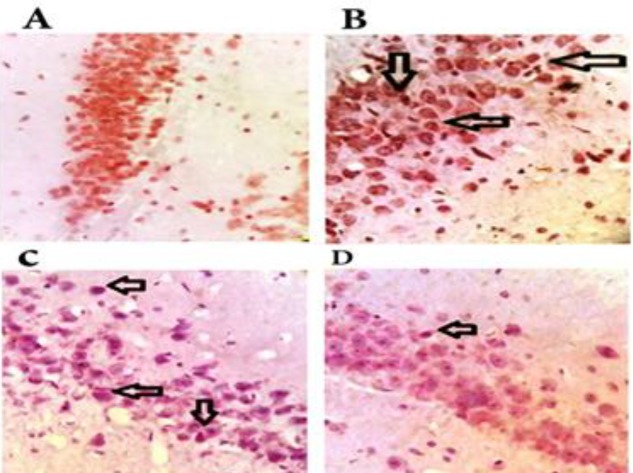
The level of caspase 3 in the hippocampus of the studied groups. A: sham (SH) group, B: chronic cerebral hypoperfusion (CCH) group, C: CCH +berberine (BBR) 50 group, D: CCH+BBR 100 group. The black arrows indicate the expression of the caspase 3 (magnification ×400)

**Figure 5:**
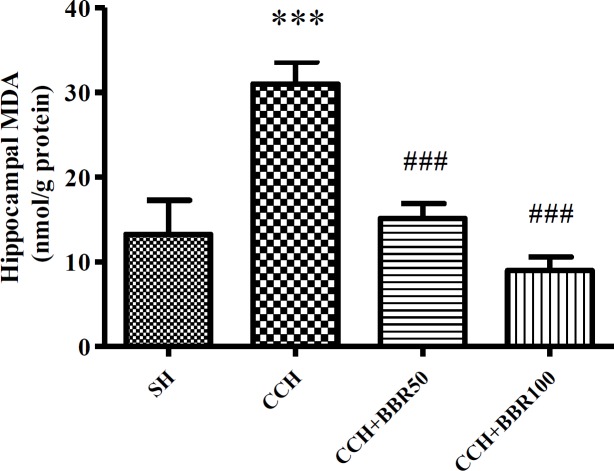
The effects of berberine (BBR) on malondialdehyde (MDA) level in the hippocampus of the studied groups. Values represent means±SEM. ***P<0.001 compared to sham (SH) group. ###*P*<0.001 compared to chronic cerebral hypoperfusion (CCH) group

**Figure 6:**
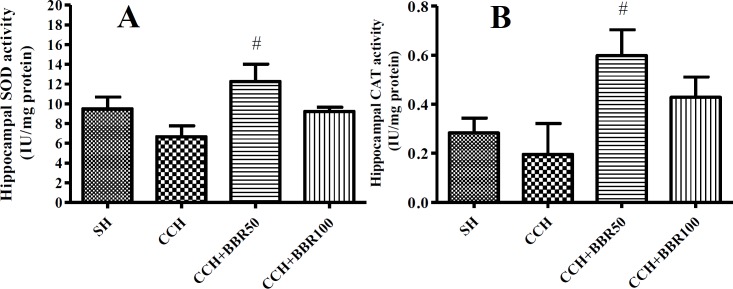
The effects of berberine (BBR) on superoxide dismutase (SOD) (Figure A) and catalase (CAT) (Figure B) activity in the hippocampus of the studied groups. Values represent means±SEM. #*P*<0.05 compared to chronic cerebral hypoperfusion (CCH) group

**Figure 7:**
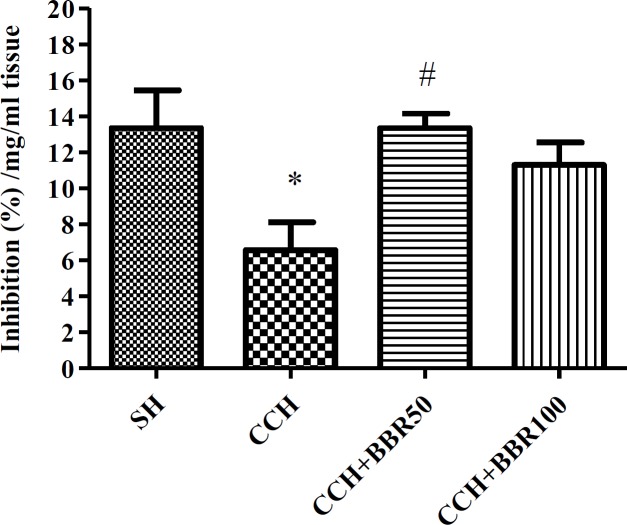
The effects of berberine (BBR) on percent inhibition of free radicals in the hippocampus of the studied groups. Values represent means±SEM. *P<0.05 compared to sham (SH) group. #*P*<0.05 compared to chronic cerebral hypoperfusion (CCH) group

***Induction of 2VO***

The permanent bilateral common carotid artery occlusion (2-vessel occlusion, 2VO) was performed to produce a model of CCH. Briefly, anesthetization of the Wistar rats was carried out by injection of xylazine (10 mg/kg) and ketamine (90 mg/kg) intraperitoneally (IP). Before anesthesia, atropine sulfate (0.1 mg/kg) was intramuscularly injected to prevent any respiratory distress. The core body temperature was measured during and after the operation and kept steady by applying a heating pad linked to a rectal probe. After creating a ventral midline incision, the bilateral common carotid arteries were revealed, which were then softly disconnected from the carotid sheath and adjacent neural bundles and quietly ligated with 3–0 silk stitch. In order to decreasing mortality rates, arterials obstruction was carried out with a seven-day interval between right and left common carotids’ occlusion. The right common carotid artery was first occluded and seven days later, the left carotid was blocked as well (15, 16). After each surgery, the cut was sewed and the animals were returned to the cages after complete removal of anesthesia. 

***Brain sample collection and histological assays***

Sixty days following occlusion, four rats from each group were made completely anesthetized with chloral hydrate (350 mg/kg, IP) then perfused with 0.9% saline in a transcardial manner and 4% paraformaldehyde in 0.1 M phosphate buffer (pH 7.4). Following that, the animals were beheaded and their brains were removed from the skull bone and fixed in the same solution for 2 days. Routine processing in paraffin were then made; briefly, the brains were dried with a rising series of ethanol, cleansed with xylene and set in paraffin, and then the brains were fixed in paraffin blocks for sectioning.

***Nissl staining***

The coronal sections with the thickness of 7 µm from hippocampus CA1 region (4 sections in each animal at 25 μm intervals) were prepared using a rotary microtome and transported to gelatinized slides. The sections dewaxed and rehydrated with xylene and ethanol and stained with 0.1% Cresyl Violet. Undamaged pyramidal neurons in CA1 area were calculated by a light microscope (400× magnification) and only pyramidal neurons with distinct nucleus and nucleoli were considered as healthy living cells (17). 

***Immunohistochemistry***

Immunohistochemical (IHC) staining was used to evaluate the activity of caspase 3. IHC was performed on 5-μm tissue sections. Briefly, 20 min were spent to incubate the tissue sections at 50 ^°^C, followed by its drying with a downward series of alcohol, and exposing with 1% hydrogen peroxide in distilled water for 5 minutes in a dark environment. To decrease the activity of endogenous peroxidase, after washing the antigens in phosphate-buffered saline (PBS) (pH=7.4), they were retrieved through autoclaving for 30 min in citrate buffer (C_6_H_5_Na3O7·2H_2_O, pH=6). Afterwards, the sections were incubated with primary antibody (ab13847) for the whole night at temperature of 4 ^°^C. Optimum dilution was reported to be 1/300. In the next stage, the tissue sections were incubated in the goat polyclonal secondary antibody (HRP) (ab97051) for one hour at room temperature by adding 3, 3′- diaminobenzidine (DAB, Dako) to obtain an image of the antigen. Finally, hematoxylin (Sigma) was added to slightly counterstain tissue sections, and the sections were then dried in alcohol, cleansed in xylene (Sigma), and mounted for imagining. The primary antibody was not used in the negative control slide’s processing. Based on the data sheet, tissue of rat lung was applied as a positive control (18).

***Brain sample collection and biochemical assays***

Sixty days following occlusion, six animals from each group were completely anesthetized with chloral hydrate (350 mg/kg, IP). The rats were decapitated and both hippocampi from each rat were quickly removed. To homogenize, the tissues were exposed with ice-cold PBS to be 10% (w/v) homogenates, which were then centrifuged at 3000 rpm at 4 ^°^C for 20 min, then the supernatants were maintained at -80 ^°^C for measurement of malondialdehyde (MDA) level in addition to superoxide dismutase (SOD), and catalase (CAT) activity. Concentration of protein was calculated using the Bradford method, where bovine serum albumin (BSA) was used as standard (19). 

***Determination of MDA in tissue***

Brain homogenates were used to determine lipid peroxidation (LPO) by response of thiobarbituric acid to MDA Assay Kit (Zellbio Co). The thiobarbituric acid reactive compound was produced following the combination of homogenate with the trichloroacetic acid, which was a red complex. The established red color of the subsequent response was estimated at 532 nm with a Nanodrape.

***Catalase activity in hippocampus tissue***

Brain homogenates were used to determine the CAT activity by using CAT assay kit (Zellbio Co) in which H_2_O_2_ is used to produce H_2_O and O_2_. H_2_O_2_ attaches to molybdenic acid to create aclathrate, which was measured at 405 nm. The H_2_O_2_ content was then calculated and expressed as μmol/mg protein.

***SOD activity in hippocampus tissue***

SOD assay kit (Zellbio Co) uses the superoxide anion for conversion to hydrogen peroxide and oxygen under enzymatic reaction conditions. SOD is one of the most effective antioxidant enzymes in the cell.

***DPPH activity in hippocampus tissue***

For DPPH activity assay, 0.0039 g of DPPH (1.1-diphenyl-2-picrylhydrazyl) powder was dissolved in 10 μl of methanol in this test. A diluent solution of 1 to 9 in methanol was obtained from this stock solution. The aforementioned diluted solution was used in this test. The microtips were taken as protocols, and the following protocols were used: 380 μl PBS (100 mM) pH 7.4 + 20 μl Sample + 400 μl DPPH. This solution was placed in a dark environment at room temperature for about 30 min and read at 530 nm using absorbance Nanodrape. The following protocol was used to use Blank: 380 µl phosphate buffer + 400 µl DPPH + 20 µl methanol. Sample absorption was calculated as follows: Activity [% of DPPH diminution] = [(A−Ax)/A]×100% (A= DPPH+ methanol, Ax= DPPH+ sample). Control A is the DPPH solution’s absorbance with methanol, and Sample A is the DPPH solution’s absorbance with tissue (20).

***Statistical analysis***

Statistical analysis was carried out using Graphpad prism 6 software. All data were analyzed using one-way analysis of variance (ANOVA) and Tukey’s *min *test was used for comparison of differences between groups. Data were presented as Mean±SEM. Statistically, differences with *P* ≤0.05 were recognized as significant.

## Results

***Berberine increased neuronal density in the hippocampus of the 2VO animals***

As presented in Figures 1 and 2, the neuronal density (the number of healthy neurons) in the hippocampal CA1 region of the CCH group was significantly lower compared to the sham group (*P*<0.001). After therapy with BBR at 50 and 100 mg/kg doses, the hippocampal neuronal density in BBR+CCH groups was considerably higher compared to the CCH group (*P*<0.01 and *P*<0.05).

***Berberine decreases caspase 3 activity in the hippocampus of the 2VO animals***

An increased activity of caspase 3 was observed in hippocampus of CCH group (*P*<0.01), while a decreased activity of caspase 3 was detected (*P*<0.05) in both treated BBR groups (Figure 3 and 4). 

***BBR increases the level of MDA in the hippocampus of the 2VO animals***

The levels of hippocampus MDA were considerably increased in the CCH group in comparison with the sham group (*P*<0.001). BBR treatment significantly attenuated MDA level in hippocampal tissue at both 50 and 100 mg/kg treatment doses (*P*<0.001) in comparison with the CCH group (Figure 5). 

***BBR increases the activities of SOD and CAT in the hippocampus of the 2VO animals***

As presented in the Figure 6, the SOD and CAT activities were reduced in the CCH group, but they were insignificant*. *BBR treatment at the dose of 50 mg/kg elevated the activities of SOD and CAT in the hippocampus of CCH-operated group (*P*<0.05); however, no significant difference was found between CCH and CCH+BBR100 groups (Figures 6A and 6B).

***DPPH scavenging effect of hippocampus tissue of rats***

Figure 7 illustrates the capability of the DPPH to scavenge homogenate hippocampus tissue. A considerable increase (*P*<0.05) was detected in the reduction of DPPH radical resulting from the scavenging ability of hippocampus tissue of BBR50-treated rats compared to the corresponding non-treated rats (Figure 7).

## Discussion

It was found that dementia due to senility and AD was linked to the lower blood flow of brain (3). Vascular dementia is the second most prevalent reason for senile dementia (21). The permanent bilateral common carotid arteries occlusion is the most common method in rats to verify the effects of CCH, and it is also used as a verified model for neurodegenerative condition and cerebrovascular insufficiency states. This ischemia model is concomitant with progressive behavioral deficits and histological changes especially in the hippocampus (3). Previous studies revealed that the conspicuous loss of neurons especially in the hippocampal CA1 subfield started 2 weeks after 2VO induction and progressed with time. An increased rate of the hippocampal destruction was observed in 8-13 weeks (3). An association was also observed between the impaired learning performance and a considerable loss of pyramidal cell in the region of CA1 (22). Numerous researches also found that the CCH-induced neuro-degeneration was accompanied with the formation of ROS that is deadly to neurons at high concentrations (23). Low levels of ROS are generated and they can be monitored via endogenous antioxidants, including CAT, glutathione peroxidase (GPx), and SOD (3). Under pathological conditions such as ischemia, ROS is overproduced, antioxidant enzymes are inactivated, and antioxidants are consumed, so that mechanisms for natural defense are unable to protect neurons from oxidative damages (24). Excessive formation of ROS can lead to the oxidization of vital parts of cells such as DNA, proteins and membrane lipids, leading to apoptotic and necrotic cell death (23). MDA is the major product of unsaturated fatty acid peroxidation as a marker of LPO. According to reports, there was a greatly important raise in the average concentration of MDA in 2VO rats after 2 month from starting surgery. Furthermore, the activity of antioxidant enzymes including GPx, SOD, and CAT reduced 2 month after the induction of 2VO in rats (3). Therefore, the therapeutic methods aiming to attenuate the oxidative stress including radical scavengers may be helpful in prolonging survival time of neurons during the cerebral ischemia (8, 24). The treatment with antioxidant has been assessed for a long duration as a tool for reducing the amount of damage caused by ischemic stroke with different levels of success (24). In another study by our group, we investigated the effects of BBR (50 mg/kg) on cognitive impairment and tissue apoptosis (16). 

In the current research, we evaluated the effect of BBR on the signaling pathway of apoptosis and oxidative stress in ischemic rats, and thus the results indicated that 2VO induction caused significant diminution in the SOD and CAT activities, and it might contribute to the occurrence of oxidative stress. We also observed a reduction of cell number in hippocampus and an increase in the LPO and caspase 3 activity in hippocampal tissue. Furthermore, the chronic administration of BBR significantly restored the changes in this tissue in the CCH group. 

BBR is a natural compound that has been safely administered to humans for long terms. There are several neuropharmacological features in BBR, including the capability of enhancing brain microcirculation and anti-neuronal apoptosis. This traditional drug has also a notable effect for preventing and inhibiting AD mostly via both beta-amyloid and cholinesterase pathways as well as its antioxidant capacities (11). 

In the present study, the MDA levels significantly reduced in BBR-treated groups, while there was an increase in the activities of CAT and SOD compared to the CCH group. Other studies were consistent with findings of the current study indicating that BBR may reduce the extreme formation of ROS and compensate antioxidant capacity (10, 25). 

Caspase 3 activity was another examined pathway in the present study. Caspase 3 activation was elevated in the group of CCH compared to the sham group. After treatment with BBR, the caspase 3 activity was nearly same to the control level. Significant evidence revealed that oxidative stress may be responsible for the apoptotic cell death caused by caspase during ischemia, and free radical scavengers decreased upregulation of caspase-3, fragmentation of DNA, and ultimate damages (26). 

## Conclusion

This was the first study suggesting that beneficial efficacy of BBR on the CCH-induced oxidative stress and apoptosis in hippocampus might be ascribed to its antioxidant feature by reducing the LPO, increasing SOD and CAT activities as well as anti-apoptotic activity by modulating the caspase-3 activity level. These remarkable findings suggested that BBR must be further assessed in terms of its medical application.

## Conflicts of Interest

The Author***s*** declares that there is no conflict of interest.
